# Mother’s satisfaction with the existing labor and delivery care services at public health facilities in West Shewa zone, Oromia region, Ethiopia

**DOI:** 10.1186/s12884-020-02998-6

**Published:** 2020-05-19

**Authors:** Gizachew Abdissa Bulto, Dereje Bayissa Demissie, Tefera Likasa Tasu, Getu Alemu Demisse

**Affiliations:** 1 Department of Midwifery, College of Medicine and Health Sciences, Ambo University, Ambo, Ethiopia; 2 Department of Neonatal Nursing, St. Paul’s Hospital Millennium Medical College, Addis Ababa, Ethiopia; 3 Department of Nursing, College of Medicine and Health Sciences, Ambo University, Ambo, Ethiopia; 4 Department of Public Health, College of Medicine and Health Sciences, Ambo University, Ambo, Ethiopia

**Keywords:** Mother’s satisfaction, Labor and delivery, Services, Public health facilities

## Abstract

**Background:**

Mothers’ satisfaction with care during childbirth is indicators of the quality care which affects skilled birth attendance. Negative client’s experiences at health facilities cause them to delay or avoid seeking care, which highlights services providers should consider and act on the expectations and experiences of women and their families. Though there are few studies conducted in Ethiopia on maternal satisfaction with Labor and Delivery (LAD) services, there is no study conducted in the study area. Therefore the study aims to assess the mother’s satisfaction with existing LAD services and associated factors at all levels of health care in the West Shewa zone.

**Methods:**

An institution-based cross-sectional study was conducted at public health facilities in West Shewa zone, Central Ethiopia. A systematic sampling technique was used to select 560 respondents by using their delivery registration number and data were collected through face to face interview. Mothers were considered satisfied if they responded satisfied/very satisfied with 75% or more of the questions assessing satisfaction. Binary and multivariable logistic regression analysis was used to identify associated factors.

**Results:**

The overall proportion of mothers who were satisfied with the current LAD care services were 60.8%. The main areas of dissatisfaction were; accessibility and cleanness of toilets/shower 72.6%, overall cleanness of the facility/including waiting-area 40.1% and presence of support a person during birth 38.0%. The presence of cultural practices (AOR = 2.5), discussion on the place of delivery with health worker during ANC (AOR = 1.75), providers asks for consent before procedure (AOR = 2.77), encouraging companion to remain with mother (AOR = 2.22), never leave mother alone or unattended (AOR = 2.56), giving periodic updates on status and progress of labor (AOR = 2.04) and explaining what is being done and to expect during LAD (AOR = 2.20) were factors identified to be significantly associated with satisfaction on LAD services.

**Conclusion:**

The overall satisfaction of mothers with LAD services at public health facilities in the West-Shewa zone was relatively low**.** Presence of cultural practices, discussion on the place of delivery, asking for consent before the procedure, encouraging companion to remain with mothers and explaining what is being done were factors identified. Therefore, all stakeholders have to emphatically work on those identified factors to improve mothers’ satisfaction with LAD services.

## Background

Globally, though there is progress on maternal mortality, an estimated 295,000 maternal deaths occurred in 2017, with an overall Maternal Mortality Ratio (MMR) of 462 in low-income countries versus 11 per 100,000 live births in high-income countries. The vast majority of deaths (94%) occurred in low-resource settings and most could have been prevented. Sub-Saharan Africa alone accounted for roughly two-thirds (196,000) of maternal deaths. Ethiopia is among the fifteen countries which were identified as very high or high alert areas of global maternal deaths by world health organization [1–3]. In Ethiopia, there has been a minimal change in MMR since 2000 [4]. According to Ethiopian demographic and health survey 2016, MMR dropped from 676 in 2011 to 412 deaths per 100,000 live births in 2016 [5].

The time of birth is critical to the survival of women and their babies [6]. Provision of good quality care during pregnancy and childbirth were among the global key intervention strategies to reduce maternal and newborn mortality. United Nations estimated that the provision of effective care for all women at the time of birth in facilities could prevent 113,000 maternal deaths by 2020 [7]. Though the institutional delivery service utilization in Ethiopia is increasing, it has remained low; 10% in 2011 and 26% in 2016 [5].

Studies suggested that providing culturally appropriate maternity care services mainly have positive effects on the use of skilled maternity care [8]. In the past interventions mainly have targeted barriers to services such as accessibility and distance to health facilities, formal or informal service charges, and lack of awareness on the benefits of skilled maternity care [2, 9–13]. Despite these interventions, many women in low and middle-income countries who had awareness on the benefits of skilled maternity care and with no other geographical barriers to access health facility continued to prefer delivering at home [10, 11, 14]. Different studies conducted in Sub-Saharan African countries identified previous negative experiences with health facilities, poor quality of care, unprofessional behavior of health workers and disrespectful care as barriers for institutional delivery services utilization [11, 12, 15–18]. Interpersonal relationships between women and health care providers were important domains of quality of care. Maternal satisfaction which is one of a quality indicator; cover all dimensions of care including; physical environment, cleanliness, availability of resources, interpersonal relationships, maintaining privacy, promptness, cognitive care, perceived provider competency, and emotional supports [19–22]. Provision of quality of maternity care services should consider and act on the expectations and experiences of women and their families [20].

Studies indicated maternal satisfaction with care provided during labor and childbirth varies in different areas; 67% in Sweden [23], 68.8% in Italy [24] and 49.4% had optimal and 29% had adequate satisfaction in Chile [25, 26]. From other studies done in Nepal (86%) [27], Bheri Zonal Hospital Nepal (89.8%) [28], Jordan 17.8% [29]*,* Egypt (78.5%) [30] and 92.5% in southern Mozambique [31] women reported as being satisfied with the care during childbirth. In Ethiopia maternal satisfaction with care during LAD ranges from 19% at St Paul Millennium Medical College Hospital to 95% among hospitals in Wolaita Zones. Different studies identified that women’s satisfaction with delivery care services was affected by their socio-demographic factors, health service quality, maternal health care utilization, obstetric factors and institutional related factors [32–41].

In Ethiopia the main reasons that have been reported for not delivering at health facilities were; health facilities were not customary, distance from facility [42], belief that it is not necessary [11], women’s previous negative experiences, lack of privacy, lack of decision making power, fear of going to an unfamiliar setting and quality of care were influencing women’s in deciding to seek care [43, 12]. While many interventions aim to improve access to skilled birth care, the quality of relationships with caregivers during maternity care has received less attention [44]. Efforts to increase the use of facility-based maternity care in low-income countries are unlikely to achieve the desired gains if there is no improvement in the quality of care provided [45]. The negative patient experiences at health facilities contribute to poor health outcomes and reinforce mistrust of institutional care which causes them to delay or avoid seeking care in health facilities [46].

Improved overall satisfaction level and quality of care during pregnancy and delivery are one of the main concerns in low and middle-income countries like Ethiopia, where the maternal mortality rate is high and yet the skilled birth attendance is very low. Though there are studies conducted on the level of maternal satisfaction with care during childbirth and associated factors most of them were limited to a single or few referrals or primary hospitals located in cities and there is no study conducted in the area. Therefore, there is a need to conduct the study on mothers’ satisfaction with care during LAD and associated factors at health facilities in West Shewa zone addressing large geographical areas and through involving rural health centers and hospitals.

## Methods

### Study design, area and period

The facility-based cross-sectional study design was conducted to assess the mother’s satisfaction with care during labor and delivery among mothers who gave birth at health facilities in West Shewa Zone from April to June 2018. The West Shewa zone has 22 districts and is located to the west of Addis Ababa. Ambo Town which is the capital of the zone is located 114 km away from Addis Ababa. According to information from the zonal health office, the total population in the zone is estimated to be 2,381,079 of which 1,214,350 of them were females. Currently, the zone has one university referral hospital, 1 general Hospital, 5 primary hospitals, 89 health centers and 447 health posts with 98% of potential health service coverage. Only public health centers and hospitals were currently providing 24 h of labor and delivery services in West Shewa zone.

### Source population and study population

All women who gave birth at public health facilities in West Shewa zone were source population and all women who gave birth at selected public health facilities during the data collection period and randomly selected using systematic random sampling were our study population. Women who were referred from other health facilities after they gave birth for different reasons were excluded.

### Sample size and sampling procedure

The sample size for the current study was determined by using a single population proportion formula with the assumptions of 79.1% proportion of mothers who were satisfied with the care they received during their childbirth in the Gamo Gofa zone, southern Ethiopia [36], 5% level of significance and 5% margin of error. Considering a 10% non-response rate and design effects of 2, the final calculated sample size was 560.

The stratified multistage sampling technique was used with the strata of the hospital and health center to select three hospitals and nineteen health centers with a simple random sampling technique by using the lottery method. Proportional allocation of study respondents was done for each selected hospitals and health centers by reviewing the number of their deliveries attended in the previous quarter (2174 deliveries). A systematic sampling technique was used to select mothers by using their delivery registration number. Every 3rd mother who gave birth at each selected health facilities were interviewed.

### Variables of the study

Mother’s satisfaction with LAD care services is the dependent variable and Independent variables were; Socio-demographic (age, marital status, religion, level of education, occupation, residence, average monthly income), obstetric related (gravidity, parity, history of institutional delivery, outcomes of previous delivery), maternal health care services and utilization (ANC, length of stay in the health facility, plan to use, mode & outcome of delivery, client-provider interactions, health facilities environment, sex of the provider, disrespect and abuses).

### Measurements of satisfaction with LAD care

The questionnaire contains six questions on physical and staff accessibility, nine on interaction with providers and staff of the facility and two on the provision of respect and privacy. The questionnaire was adapted from previously done similar studies in Ethiopia, which have adopted their tools from Donabedian quality assessment framework [21, 32, 34, 36, 38] (Additional files 1). Responses were recorded with a five-point Likert scale (very dissatisfied, dissatisfied, neutral or undecided, satisfied and very satisfied) for all seventeen questions. The reliability of the used questions was checked with Cronbach’s alpha, which was found to be 0.81. Mothers were considered satisfied with the care received from health facilities if they responded satisfied or very satisfied to 75% or more of 17 questions assessing maternal satisfaction which was determined based on previous studies conducted in Ethiopia [32, 34, 36, 38, 41].

### Data collection tools and techniques

The questionnaire was initially prepared in English and translated into Afan Oromo (local language) and back to English to check for its consistency with language experts. Data was collected through face to face interview by using the Afan Oromo version questionnaire, after the health care provider’s decision to discharge the mothers but before they left health facilities. Data were collected in a private setting to let them respond freely for questions. Twenty-four Nurses who were not working in the selected health facilities collected data and three Masters level supervisors have conducted supervision during the data collection period. Training on the objectives and techniques of data collection was given for data collectors for two days one week before the actual data collection period. Pre-test of the questionnaire was done on 5% of the women who delivered at Holeta health center and Inchini hospital and necessary corrections were made accordingly. Daily supervision was conducted by supervisors during data collection.

### Data processing and analysis

The questionnaires were checked for completeness, coded and entered into EPI Data version 3.1software and then exported to SPSS windows version 23 for analysis. Bivariate analysis was used primarily to check variables which have an association with the dependent variable individually. Hosmer and Lemeshow goodness of fit test was checked, and it was found to be 0.989 on the final model. Variables which were found to have an association with the dependent variable at a *P*-value of < 0.2 were then entered into multivariable logistic regression for controlling the possible effect of confounders and finally the variables which have significant association were identified based on AOR, with 95%Confidence Interval (CI) and *P*-value < 0.05.

### Ethical considerations

Ethical clearance was obtained from the research review and ethics committee of the College of Medicine and Health Sciences, Ambo University with the reference number of AU/CMHSRCS/59/2018.

## Results

In this study, a total of 558 mothers fully responded, making a response rate of 99.64%. The mean age of the mothers was 26.92 with a standard deviation of 5.21 years and almost half 46.6% attended at a health center. A third of them were housewives 35.5 and 26.7% of them had primary education. The majority of respondents were married 96.2%, belongs to Oromo ethnic group 93.9% and from urban residence 69.5% (Table 1).

**Table 1:** A Socio-Demographic characteristic of mothers who gave birth at public health facilities in west Shewa zone, Oromia region, Ethiopia, 2018

Characteristics	Categories	Number (***N*** = 558)	Percent (%)
Type of Institution	Health centers	260	46.6
	Primary Hospitals	153	27.4
	General Hospital	145	26.0
Age in years	17–24	189	33.9
	25–29	202	36.2
	More than 29	167	29.9
Marital Status	Married	537	96.2
	Other marital Status^1^	21	3.8
Religion	Orthodox	218	39.1
	Protestant	301	53.9
	Other Religion^2^	39	7.0
Ethnicity	Oromo	524	93.9
	Other Ethnicity^3^	34	6.1
Educational Status	Have no formal education	143	25.6
	Primary education	149	26.7
	Secondary Education	130	23.3
	Collage and above	136	24.4
Mothers Occupation	Government Employee	122	21.9
	Housewife	198	35.5
	Farmers	84	15.1
	Merchant	75	13.4
	Private Employee’	66	11.8
	Other Occupation^4^	13	2.3
Mothers Residence	Urban	388	69.5
	Rural	170	30.5
Average Monthly Income (Interquartile range) with Ethiopian Birr	Less than 1000	140	25.1
	1001–2000	150	26.9
	2001–3500	132	23.7
	Greater than 3500	136	24.4
	Median = 2000.00		

Keys: ^1^Divorced, widowed & single, ^2^Wakefata & Catholic, ^3^Gurage & Tigre, ^4^Student & Daily laborers

### Obstetric characteristics of mothers who gave birth in west Shewa zone

In this study majority of mothers were multiparas 73.7%, gave birth with spontaneous vaginal delivery 79.9%, gave birth without any complications 87.8% and were attended by Midwives 74.2% (Table 2).

**Table 2:** Obstetric characteristics of mothers who gave birth at public health facilities in west Shewa zone, Oromia region, Ethiopia, 2018

Characteristics	Categories	Number (*N* = 558)	Percent (%)
Ever given Birth before	Yes	411	73.7
	No	147	26.3
Number of Parity	Primi-para	147	26.3
	2–3	207	37.1
	> 4	204	36.6
Had ANC follow-ups	Yes	528	94.6
	No	30	5.4
Number of ANC follow-ups	No ANC follow-up	30	5.4
	Once to twice	90	16.1
	Three times	173	31.0
	Four and above	265	47.5
HCPs discussed the place of delivery during ANC	Yes	453	81.2
	No	105	18.8
Discussed place of delivery with partner	Yes	400	71.7
	No	158	28.3
Duration spent on labor	Less than or equal to 12 h	410	73.5
	More than 12 h	148	26.5
Mode of Delivery	Spontaneous vaginal delivery	446	79.9
	Assisted vaginal Delivery	44	7.9
	Cesarean section	68	12.2
Condition of the mother during childbirth	No Complications	490	87.8
	Had Complications	68	12.2
Time of delivery	Day time	298	53.4
	Night Time	260	46.6
Who attended your delivery	Midwife	414	74.2
	Nurse or Health Officer	38	6.8
	Doctors or Emergency Surgeon	106	19.0
Asked your consent before performing procedures	Not asked me	79	14.2
	Yes	235	42.1
	No Procedure is done for me	244	43.7
Presence of cultural practices for mothers (Coffee or porridge)	Yes	504	90.3
	No	54	9.7
Admitted to maternity waiting home before labor started	Yes	32	5.7
	No	526	94.3

### Mothers satisfaction with labor and delivery care services in west Shewa zone

In this study, the proportion of mothers who were satisfied with the LAD care services received from health facilities in West Shewa zone was 339(60.8%) (95% CI: 56.5, 64.9). Technical competency of the provider 94.1%, waiting time to be seen by health worker 87.3%, Health care providers (HCPs) were helpful during labor and/or birth 92.3% and providers use of appropriate/friendly language 93.7% were those elements on which mothers were highly satisfied. Whereas the main causes of dissatisfaction identified were; accessibility and cleanness of toilets/shower 72.6%, overall cleanness of the facility/including waiting-area 40.1%, examination area cleanliness and comfort 35.8%, presence of a support person during birth 38.0% and delivery position of choice 49.6% of them were dissatisfied (Tables 3 and 4).

**Table 3:** Mother’s satisfaction with care during labor and delivery at public health facilities in West Shewa Zone, Oromia region, Ethiopia, 2018

Categories	Variables	Satisfied with LAD care	
		Yes ***N*** (%)	No ***N*** (%)
**Physical and Staff accessibility**	Examination area cleanliness and comfort	358(64.2)	200(35.8)
	Overall cleanness of the facility/including waiting area	334(59.9)	224(40.1)
	Accessibility and cleanness of toilets/shower	153(27.4)	405(72.6)
	Availability of supplies basic drugs and equipment	429(76.9)	129(23.1)
	Technical competency of care providers	525(94.1)	33(5.9)
	Waiting time to be seen by the health worker	487(87.3)	71(12.7)
**Interaction with Providers and Staff of the Health Facility**	HCP were friendly and welcoming on arrival	477(85.5)	81(14.5)
	HCPs were encouraging and reassuring during LAD	457(81.9)	101(18.1)
	HCPs were helpful during labor and/or birth	515(92.3)	43(7.7)
	Overall care during labor and birth was good	505(90.5)	53(9.5)
	HCP kept you informed what was happening during LAD	351(62.9)	207(37.1)
	Involvement of patient in decision making	472(84.6)	86(15.4)
	Presence of support person during birth	346(62.0)	212(38.0)
	Confidentiality and trust in providers	458(82.1)	100(17.9)
	Delivery position of patient choice	281(50.4)	277(49.6)
**Provision of Respect and Privacy**	Respect and assurance of privacy during exam and delivery	489(87.6)	69(12.4)
	Providers used appropriate/friendly language	523(93.7)	35(6.3)
Overall satisfaction with labor and delivery care services received from health facilities in West Shewa zone		339(60.8%)	219(39.2)

**Table 4:** Satisfaction with care during LAD with some socio-demographic and obstetric characteristics among mothers’ who gave birth at public health facilities in West Shewa Zone, Oromia region, Ethiopia, 2018

Characteristics	Categories	Dissatisfied with LAD care (%)	Satisfied with LAD care (%)
Type of Institution	Health centers	104 (40.0)	156 (60.0)
	Primary Hospitals	57 (37.3)	96 (62.7)
	General Hospital	58 (40.0)	87 (60.0)
Age in years	17–24	71 (37.6)	118 (62.4)
	25–29	80 (39.6)	122 (60.4)
	More than 29	68 (40.7)	99 (59.3)
Marital Status	Married	211 (39.3)	326 (60.7)
	Other marital Status^1^	8 (38.1)	13 (61.9)
Religion	Orthodox	70 (32.1)	148 (67.9)
	Protestant	132 (43.9)	169 (56.1)
	Other Religion^2^	17 (43.6)	22 (56.4)
Ethnicity	Oromo	210 (40.1)	314 (59.9)
	Other Ethnicity^3^	9 (26.5%)	25 (73.5)
Educational Status	Have no formal education	60 (42.0)	83 (58.0)
	Primary education	73 (49.0)	76 (51.0)
	Secondary Education	41 (31.5)	89 (68.5)
	Collage and above	45 (33.1)	91 (66.9)
Mothers Occupation	Government Employee	41 (33.6)	81 (66.4)
	House wife	89 (44.9)	109 (55.1)
	Farmers	30 (35.7)	54 (64.3)
	Merchant	27 (36.0)	48 (64.0)
	Private Employee’	25 (37.9)	41 (62.1)
	Other Occupation^4^	7 (53.8)	6 (46.2)
Mothers Residence	Urban	152 (39.2)	236 (60.8)
	Rural	67 (39.4)	103 (60.6)
Parity	Primi-para	59 (40.1)	88 (59.9)
	2–3	80 (38.6)	127 (61.4%)
	> 4	80 (39.2)	124 (60.8%)
Had ANC follow-ups	Yes	204 (38.6)	324 (61.4)
	No	15 (50.0)	15 (50.0)
Duration spent on labor	Less than/equal to 12 h	187 (45.6)	223 (54.4)
	More than 12 h	32 (21.6)	116 (78.4)
Mode of Delivery	Spontaneous vaginal delivery	187 (41.9)	259 (58.1)
	Assisted vaginal Delivery	12 (27.3)	32 (72.7)
	Cesarean section	20 (29.4)	48 (70.6)
Outcomes of delivery	Alive	215 (39.8)	325 (60.2%)
	Dead	4 (22.2)	14 (77.8)
Condition of mother during childbirth	No Complications	198 (40.4)	292 (59.6)
	Had Complications	21 (30.9)	47 (69.1)
Time of delivery	Day time	101 (33.9)	197 (66.1)
	Night Time	118 (45.4)	142 (54.6)

Keys: ^1^divorced, widowed & single, ^2^Wakefata & Catholic, ^3^Gurage & Tigre, ^4^Student & Daily laborers

### Factors associated with mothers’ satisfaction with care during labor and delivery

On bivariate analysis outcome of delivery, provision of comfort/pain relief as necessary, asking for consent before the procedure, presence of cultural practices in the health facilities, speaking politely to women and companion, encouraging a companion to remain with women, time or shift of delivery, discussion on the place of delivery with partner, never insulted or intimidated, explain what is being done and expect throughout LAD, giving periodic updates on status and progress, admitted to maternity waiting home before labor starts, no disrespect based on specific attribute, never leave women alone or unattended and discussing the place of delivery with health care provider during ANC follow-ups were associated with mothers satisfaction on care during LAD at *P*-value of < 0.2.

On multivariable logistic regression analysis presence of cultural practices, discussion on the place of delivery with health worker during ANC, asked for consent before the procedure, encouraging companion to remain with mother’s, never leave mother alone or unattended, giving periodic updates on status and progress of labor and explaining what is being done and to expect during LAD were factors identified to be significantly associated with mothers’ satisfaction with care during LAD.

This study identified that those mothers who gave birth at facilities where there are cultural practices like coffee and porridge were 2.5 times more likely to be satisfied than those where there is no (AOR = 2.5, 95% CI: 1.28, 4.94) and those who had discussed on the place of delivery with health worker during ANC were almost two times more likely to be satisfied than those who did not (AOR = 1.75, 95% CI: 1.03, 2.96).

The current study also identified that those mothers who were asked for consent before performing the procedure and whom no procedure was done were 2.77 and 1.9 times more likely to be satisfied with LAD service provided than those who had the procedure done without their consent (AOR = 2.77, 95% CI: 1.43,5.35) and (AOR = 1.90, 95% CI: 1.01, 3.57) respectively.

In this study, those mothers who gave birth at areas where the health workers encourage a companion to remain with them were twice more likely to be satisfied with the care received during LAD than those who were not (AOR = 2.22, 95% CI: 1.42, 3.46). Those mothers who received periodic updates on the status and progress of their labor from care providers during LAD were twice more likely to be satisfied than those who were not (AOR = 2.04, 95% CI: 1.30, 3.20) (Table 5).

**Table 5:** Factors associated with Mothers’ satisfaction with care during labor and delivery at health Facilities in West Shewa Zone, Oromia region, Ethiopia, 2018

Variables	Satisfied with care during LAD		Crude OR 95% CI	Adjusted OR 95% CI	***P***-value
	Yes	No			
Presence of cultural practices (Coffee or Porridge)					
Yes	45(83.3)	9(16.7)	3.57(1.70, 7.46)	2.51(1.28,4.94)	
No	294(58.3)	210(41.7)	1	1	0.007
Discussed on the place of delivery during ANC					
Yes	301(66.4)	152(33.6)	3.49(2.24,5.43)	1.75(1.03,2.96)	0.036
No	38(36.2)	67(63.8)	1	1	
Asked for consent before performing procedures					
Not asked me	28(35.4)	51(64.6)	1	1	0.008
Yes	164(69.8)	71(30.2)	4.20(2.45, 7.20)	2.77(1.43,5.35)	0.002
No procedure is done	147(60.2)	97(39.8)	2.76(1.62, 4.67)	1.90(1.01,3.57)	0.045
Encourage companion to remain with mother’s					
Yes	271(70.9)	111(29.1)	3.87(2.66, 5.64)	2.22(1.42,3.46)	
No	68(38.6)	108(61.4)	1	1	0.0004
Never leave women alone or unattended					
Yes	258(74.4)	89(25.6)	4.65(3.22, 6.71)	2.56(1.61,4.06)	
No	81(38.4)	130(61.6)	1	1	0.00006
Gives periodic updates on status and progress of labor					
Yes	273(71.7)	108(28.3)	4.25(2.91, 6.20)	2.04(1.30,3.20)	
No	66(37.3)	111(62.7)	1	1	0.002
Explain what is being done and to expect during LAD					
Yes	261(75.2)	86(24.6)	5.17(3.57, 7.49)	2.20(1.39,3.47)	
No	78(37.0)	133(63.0)	1	1	0.001

## Discussion

Maternal satisfaction includes all dimensions of care including; physical environment, cleanliness, availability of resources, interpersonal behavior, privacy, promptness, cognitive care, perceived provider competency, and emotional supports. In this study, the proportion of mothers who were satisfied with the LAD care services they received from health facilities in West Shewa zone was 60.8% (95% CI: 56.5, 64.9). This is in-line with the study done at Referral hospitals in Amhara region (61.9%) [32].

This finding is higher than studies conducted at Health facilities in Mizan Aman Town among mothers who gave birth in the last one year (30.4%) [37], St. Paul’s Hospital Millennium Medical College Addis Ababa (19%) [39] and Jordan 17.8% [29]. The possible reason for this might be due to the difference in a study setting in which the current study was conducted at health centers and hospitals located in small towns and rural parts of the country where the clients’ awareness and expectations of health services provided were low compared to those from a big city like Addis Ababa. Also, evidence indicates mother’s satisfaction with care during childbirth was lowered with time, which is in the case of Mizan Aman [37] and Jordan’s [29]; and St. Paul’s Hospital is a specialized teaching hospital which has many caseloads than institutions in the current study area.

The current study finding is lower than studies done at Felege Hiwot Referral Hospital Bahir Dar (74.9%) [33], Debre Markos among mothers who gave birth within one year (81.7%) [34], Asrade Swede Memorial Primary Hospital West Gojjam (88%) [35], Hospitals in Wolaita Zone (95%) [47], Gamo Gofa Zone (79.1%) [36], Harari region (84.7%) [38], Asella hospital (80.7%) [40], health facilities in West Arsi Zone (74.6%) [41], Southern Mozambique (92.5%) [31] and Bheri Zonal Hospital, Nepal (89.88%) [28]. These variations might be due to most of those studies were conducted at hospitals where there are relatively adequate infrastructure, supplies and human resources with the presence of a mix of professionals, including specialty care in those study areas, but the current study was conducted at health centers and hospitals located more in small towns and in rural parts of the country. It is also due to the differences in the quality of services provided in those study areas specifically Felege Hiwot, Asella, Wolaita and hospitals in Harar. Studies in Wolaita and Harar also included private hospitals that were providing quality services than government institutions.

Health centers and hospitals working environment is the main source of dissatisfaction in the study area. The main areas of dissatisfaction in the current study were; accessibility and cleanness of toilets or shower rooms, overall cleanness of the facility/including waiting area, examination area cleanliness, and comfort, presence of a support person during birth and delivery position of choice. In line with these previous studies also indicated; overall cleanliness and comfort of examination rooms and waiting areas, cleanliness and access to the toilets, and availability of hand-washing facility and shower were the main areas of dissatisfaction [35, 36, 40, 41, 47].

The provision of culturally sensitive quality health services is important for enhancing maternal health services utilization. Those mothers who gave birth at facilities where there is a cultural practice like coffee and porridge were 2.5 times more likely to be satisfied than those where there are no practices. The reason might be due to those practices that were common to do in the area immediately after the mother gave birth at home, therefore, those mothers who get an opportunity to practice were more likely to feel the health institutions like their home.

Those who had discussed the place of delivery with health workers during ANC were 1.75 times more likely to be satisfied than those who did not. In line with this, a study done at Debre Markos showed that having a plan to deliver at health institutions was 3 times more likely to be satisfied than those who did not [34]. This might be due to those mothers who had discussed the place of delivery during ANC were likely to receive education on birth preparedness and complication readiness plan and at some institutions providers let the pregnant mother visit their LAD room to familiarize them.

Requesting for consent before any procedure is found to be an important factor affecting maternal satisfaction with LAD care services. Those women who were asked for consent before performing the procedure and those whom no procedure was done were 2.77 and 1.9 times more likely to be satisfied with LAD service provided than those who had a procedure done without asking their consent. The possible explanation for this might be those mothers whom the provider did not take consent before the procedure was likely to perceive as the provider undermined them and feel as they did not consider their right for engagement in their care decision making.

Evidence suggests companionship of choice during labor and childbirth may increase spontaneous vaginal birth, reduces cesarean section and instrumental vaginal births [48]. The world health organization also recommended a companion of choice for all women throughout labor and childbirth [6]. In this study, those mothers who gave birth at institutions where the health workers encourage companion to remain with mothers were twice more likely to be satisfied with the care received during LAD than those who were not. In agreement with this study done in southern Mozambique has shown having a companion during childbirth increased the overall satisfaction of mothers [31] and in Gamo Gofa Zone the presence of support persons during childbirth was significantly associated with institutional delivery service satisfaction [36].

Women who were provided with continuous support during labor and childbirth were at increased chance of spontaneous vaginal birth, had no harm, and were more satisfied [48]. Mothers who were provided uninterrupted care during LAD from care providers were 2.56 times more likely to be satisfied with the care they received than those who were left alone or unattended. Similarly, the study done in southern Mozambique showed that mothers who experienced as being abandoned when needing help reported low levels of satisfaction [31], and in Harar Hospitals participants who waited for 15 min or less to be seen by the HCPs were 2.5 times more likely to be satisfied compared to women who waited for more [38].

The study revealed that those mothers whom health workers provided periodic updates on the status and progress of their labor from care providers during LAD were twice more likely to be satisfied than those who were not and those who gave birth at areas where care providers explain what is being done and to expect during LAD were twice more likely to be satisfied than those who were not provided. Likewise, studies done in Nepal indicated having an opportunity to ask questions was positively associated with client satisfaction [49], at referral Hospitals in Ethiopia inadequate information about the drugs and explanation of procedures to be done to the client were found to be major causes of dissatisfaction [50], St. Paul Hospital Addis Ababa shown that the women who had an opportunity to talk with health care providers were 2.44 times satisfied than those who did not [39], and at Asrade Zewude Memorial primary hospital mothers who care providers listened and answered their questions during delivery were more likely to be satisfied than those who did not [35].

### Limitations and strength

The study is not free from social desirability bias since we have collected data at health facilities with an exit interview and being cross-sectional as it is difficult to assess temporal relationships were some of the limitations of this study. Addressing a larger number of health facilities both involving health centers and hospitals might be the strength of this study compared to other previous studies.

## Conclusion

The study demonstrated the overall satisfaction of mothers with LAD care services they received from public health facilities in West Shewa zone was relatively low. The study identified the presence of cultural practices, discussion on place of delivery with health worker during ANC, asking for consent before the procedure, encouraging companion to remain with mother, never leaving the mother alone or unattended, giving periodic updates on status and progress of labor and explaining what is being done and to expect during LAD were factors identified to be significantly associated with mothers satisfaction on care during LAD.

Therefore, the federal ministry of health, regional health bureau, zonal health office and health facilities should have to work on making health facilities working environments more conducive or comfortable for the clients through maintaining the cleanliness of the examination and waiting-areas and making clean toilets, shower rooms accessible for the mothers in all health facilities.

Health facilities and health workers should work on promoting cultural practices in their health facilities, encourage the presence of a companion, providing uninterrupted care, requesting for consent before any procedure, and give periodic updates on the status and progress of labor during LAD is recommended. Further study is also recommended to explore on cultural preferences of mothers during labor and childbirth which might affect their delivery preference and satisfaction level.

## Supplementary information

**Additional file 1.** English version Questionnaire

## Data Availability

Datasets used in the current study are available from the corresponding author on reasonable request.

## Abbreviations

ANC: Ante Natal Care
AOR: Adjusted Odds Ratio
HCPs: Health care Professionals
LAD: Labor And Delivery
MMR: Maternal Mortality Ration
PNC: Post Natal Care
