# Supplementary material for: Mother’s satisfaction with the existing labor and delivery care services at public health facilities in West Shewa zone, Oromia region, Ethiopia
Source: BMC Pregnancy Childbirth. 2020 May 19;20:303. doi: 10.1186/s12884-020-02998-6 (PMC7236095; doi:10.1186/s12884-020-02998-6)
Supplement: Supplementary file 1 — Additional file 1. English version Questionnaire [file 12884_2020_2998_MOESM1_ESM.docx]

## 1: English version Questionnaire

**Background Information**

1. Type of Health Institution
   1. Health Center
   2. District Hospital
   3. General Hospital

**Part I: Socio-demographic characteristics**

| **No.** | **Questions** | **Response** | **Remark** |
| --- | --- | --- | --- |
| 101 | Age in years | __________years |  |
| 102 | Marital status | 1. Married  2. Divorced  3. Widowed  4. Others (specify)_________ |  |
| 103 | Religion | 1. Orthodox  2. Protestant  3. Muslim  4. Catholic  5. Others (specify)__________ |  |
| 104 | Ethnicity | 1. Oromo 2. Amhara 3. Gurage 4. Others (Specify) _________ |  |
| 105 | Educational status | 1. Unable to read and write  2. Read and Write  3. Primary education(1-8)  4. High school(9-12)  5. Diploma and above |  |
| 106 | Occupation status | 1. Government employee 2. House wife 3. Farmers 4. Merchant 5. Private Employee   6. Others (specify)___________ |  |
| 107 | Residence | 1.Urban  2. Rural |  |
| 108 | Average Monthly family income in Birr | _______________ Birr |  |
| 109 | Access to media sources (As a source of information about maternal health care services) ***Choose all that applies*** | 1.TV  2. Radio  3. Newspaper, Magazine  4. Others (Specify) ________ |  |

**Part-II: Maternal Health care and services related questions**

| **No.** | **Questions** | **Response** | | | **Remark** | |
| --- | --- | --- | --- | --- | --- | --- |
| 201 | Have you ever given birth before? | 1. Yes  2. No | | | If No SKIP to ----- 203 | |
| 202 | How many children have you given birth (Parity)? | ___________ Children | | |  | |
| 203 | Is the current pregnancy based on need or wanted? | 1. Yes wanted  2. Not wanted | | |  | |
| 204 | Do you have ante natal care follow up for current pregnancy? | 1. Yes 2. No | | |  | |
| 205 | How many times you had Antenatal care follow up? | ____________ (Number of ANC visits) | | |  | |
| 206 | Where have you attended your Ante natal care follow up for current pregnancy? ***(Choose all that apply)*** | 1. Health Center 2. Hospital 3. Health post 4. Private clinics | | |  | |
| 207 | Did health care providers discussed about places of delivery? | 1.Yes  2. No | | |  | |
| 208 | Have you discussed about your current place of delivery with your partner? | 1. Yes 2. No | | |  | |
| 209 | Who primarily accompanied you to this health institution for delivery? | 1.Husband  2. My mother  3. mother in law  4. Neighbors  5. Others specify ____________ | | |  | |
| 210 | Health Center or Hospital visit type for current delivery. | 1. New (my 1^st^ time)  2. Repeat visits  3. Referred from other institution | | |  | |
| 211 | How did your labor started? | 1.Spontaneous  2. Induced | | |  | |
| 212 | Total duration you spent on labor. | _____________ hours | | |  | |
| 213 | Duration of stay at current health institution before you gave birth? | ___________ hours | | |  | |
| 214 | Duration of stay at current health institution after you gave birth? | __________ hours | | |  | |
| 215 | Type or mode of delivery. | 1. Spontaneous vaginal delivery 2. Assisted vaginal delivery (vacuum or Forceps) 3. Cesarean section 4. Others specify____________ | | |  | |
| 216 | Out-comes of delivery. | 1. Alive 2. Already dead before arrival 3. Dead after delivery | | |  | |
| 217 | Conditions of the mother during and after child birth. | 1.Normal  2. Had complications | | |  | |
| 218 | Time of delivery (During which ***Shift***) | 1. Day time 2. Night time | | |  | |
| 219 | Who attended your delivery or provided you the service? | 1.Midwife  2. Nurse  3. Health Officers  4. Medical Doctor  5. Other (Specify)___________ | | |  | |
| 220 | Sex of the health provider who attended you during child birth. | 1.Male  2.Female | | |  | |
| 221 | Do they have done any procedure for you? (***Choose all that apply***) | 1. Episiotomy 2. Fundal pressure 3. Manual removal of placenta 4. Instrumental delivery 5. Cesarean section 6. Others (specify) ___________ | | |  | |
| 222 | Did they have asked your consent before performing this procedure? | 1. Not asked me 2. Yes 3. Yes but done with-out my will 4. No procedure was done for me | | |  | |
| 223 | Encourages companion to remain with woman whenever possible |  | | |  | |
| 224 | Explains what is being done and what to expect throughout labor and birth (Tubal ligation, episiotomy, abdominal palpation, vaginal examination, others) |  | | |  | |
| 225 | Gives periodic updates on status and progress of labor (Explained finding of general examination) |  | | |  | |
| 226 | Never leaves woman alone or unattended (provided continuous support during labor) |  | | |  | |
| 227 | Does not show disrespect to women based on any specific attribute |  | | |  | |
| 228 | Never uses physical force or abrasive behavior with the woman, including slapping, hitting, pinching, roughly forcing legs apart, fundal pressure for normal delivery, pushed |  | | |  | |
| 228 | How many health workers or individuals attending you during child birth? | ___________ in number | | |  | |
| 229 | **Maternity Waiting Home services** | | | | | |
| **a.** | Does Maternity waiting home available? | | 1.Yes | 2. No | |  |
| **b.** | Does coffee ceremony available at this institution? | | 1.Yes | 2. No | |  |
| **c.** | Is porridge services is available at this health institution? | | 1.Yes | 2. No | |  |
| **d.** | Are you admitted to maternity waiting homes before the labor started? | | 1.Yes | 2. No | |  |
| 230 | **How many times you delivered at health institution? (*Including the current one*)** | | --------------------. | |  | |
| 231 | **Do you want to have a child in the future?** | | 1.Yes  2.No | | **If No skip to 230** | |
| 232 | **If yes** do you want to deliver in the same facility for the future? | | 1. Yes 2. No | |  | |
| 233 | Do you recommend this health facility for others to deliver here? | | 1. Yes 2. No | |  | |
| 234 | **If no** to question number **229** why you don’t want to deliver at the same facility? | | _______________________________ . | | | |

**Part III: Questionnaires Assessing Maternal Satisfaction on Labor and Delivery Services**

| **S.No** | **Labor and Delivery Satisfaction Index** | **Responses** | | | | | **Comment** |
| --- | --- | --- | --- | --- | --- | --- | --- |
|  |  | **Very unsatisfied** | **Unsatisfied** | **Undecided /Neutral** | **Satisfied** | **Very satisfied** |  |
| **Physical and staﬀ accessibility** | |  |  |  |  |  |  |
|  | Examination area cleanliness and comfort |  |  |  |  |  |  |
|  | Overall cleanness of the facility /including waiting area |  |  |  |  |  |  |
|  | Accessibility and cleanness of toilets and/or shower |  |  |  |  |  |  |
|  | Availability of supplies: basic drugs and equipment |  |  |  |  |  |  |
|  | Technical competency of care providers |  |  |  |  |  |  |
|  | Waiting time to be seen by health worker |  |  |  |  |  |  |
| **Interaction with providers and staﬀ of the facility** | |  |  |  |  |  |  |
|  | Health care provider were friendly and welcoming when you arrived at the hospital |  |  |  |  |  |  |
|  | Health care providers were encouraging and reassuring during labor and/or birth. |  |  |  |  |  |  |
|  | Health care providers were helpful during labor and/or birth. |  |  |  |  |  |  |
|  | The overall care during labor and birth was good |  |  |  |  |  |  |
|  | The health care provider always kept you informed about what was happening during labor & birth. |  |  |  |  |  |  |
|  | Involvement of patient in decision making |  |  |  |  |  |  |
|  | Presence of support person during birth |  |  |  |  |  |  |
|  | Confidentiality and trust in providers |  |  |  |  |  |  |
|  | Delivery position of patient choice |  |  |  |  |  |  |
| **Provision of respect and privacy** | |  |  |  |  |  |  |
|  | Respect and assurance of privacy during examination and delivery |  |  |  |  |  |  |
|  | The providers who attended to you used appropriate/friendly language |  |  |  |  |  |  |

**The end**

**Thank you for your time and co-operation!**
